# Glycogen synthase kinase 3 beta inhibits microRNA-183-96-182 cluster via the β-Catenin/TCF/LEF-1 pathway in gastric cancer cells

**DOI:** 10.1093/nar/gkt1275

**Published:** 2013-12-12

**Authors:** Xiaoli Tang, Dong Zheng, Ping Hu, Zongyue Zeng, Ming Li, Lynne Tucker, Renee Monahan, Murray B. Resnick, Manran Liu, Bharat Ramratnam

**Affiliations:** ^1^Division of Infectious Diseases, Department of Medicine, Warren Alpert Medical School of Brown University, Providence, R I02903, USA, ^2^Laboratory of Genetics and Molecular Biology, Division of Physiology, Department of Zoology, Northeast Forestry University, Harbin 150040, China, ^3^Key Laboratory of Laboratory Medical Diagnostics, Chinese Ministry of Education, Chongqing Medical University, Chongqing 400016, China and ^4^Department of Pathology, Warren Alpert Medical School of Brown University, Providence, RI02903, USA

## Abstract

Glycogen synthase kinase 3 beta (GSK3β) is a critical protein kinase that phosphorylates numerous proteins in cells and thereby impacts multiple pathways including the β-Catenin/TCF/LEF-1 pathway. MicroRNAs (miRs) are a class of noncoding small RNAs of ∼22 nucleotides in length. Both GSK3β and miR play myriad roles in cell functions including stem cell development, apoptosis, embryogenesis and tumorigenesis. Here we show that GSK3β inhibits the expression of miR-96, miR-182 and miR-183 through the β-Catenin/TCF/LEF-1 pathway. Knockout of GSK3β in mouse embryonic fibroblast cells increases expression of miR-96, miR-182 and miR-183, coinciding with increases in the protein level and nuclear translocation of β-Catenin. In addition, overexpression of β-Catenin enhances the expression of miR-96, miR-182 and miR-183 in human gastric cancer AGS cells. GSK3β protein levels are decreased in human gastric cancer tissue compared with surrounding normal gastric tissue, coinciding with increases of β-Catenin protein, miR-96, miR-182, miR-183 and primary miR-183-96-182 cluster (pri-miR-183). Furthermore, suppression of miR-183-96-182 cluster with miRCURY LNA miR inhibitors decreases the proliferation and migration of AGS cells. Knockdown of GSK3β with siRNA increases the proliferation of AGS cells. Mechanistically, we show that β-Catenin/TCF/LEF-1 binds to the promoter of miR-183-96-182 cluster gene and thereby activates the transcription of the cluster. In summary, our findings identify a novel role for GSK3β in the regulation of miR-183-96-182 biogenesis through β-Catenin/TCF/LEF-1 pathway in gastric cancer cells.

## INTRODUCTION

Glycogen synthase kinase 3 beta (GSK3β) is a serine/threonine protein kinase whose function is required for the NF-kB–mediated anti-apoptotic response to tumor necrosis factor alpha (1). GSK3β also plays a critical role in numerous signaling pathways including Wnt/β-Catenin/TCF/LEF-1 signaling pathway. GSK3β is constitutively active in cells and forms a complex with adenomatous polyposis coli (APC) and scaffold protein Axin in the absence of Wingless/Wnt signal. Phosphorylation of APC by GSK3β provides a docking site for β-Catenin binding. β-Catenin is a key component of both the cadherin cell adhesion system and the Wnt signaling pathway (2–4). GSK3β phosphorylates β-Catenin leading to its degradation by ubiquitin-proteasome pathway (5). Wnt signal inhibits GSK3β activity and increases free cytosolic β-Catenin level. β-Catenin translocates to the nucleus to act as a cofactor for the T cell factor (TCF) family of transcription factors, including TCF-1, TCF-3, TCF-4 and LEF-1 (leukemia enhancer factor 1). β-Catenin/TCF/LEF-1 complex activates oncogenic target genes such as c-myc (6), c-jun (7) and cyclin D1 (8).

Our previous studies showed that GSK3β phosphorylates Drosha, the key RNase III enzyme that initiates microRNA (miR) biogenesis (9,10). MiRs are transcribed into primary miRs (pri-miRs) from miR genes by polymerase II or III. Pri-miRs are processed into shorter precursor miRs (pre-miRs) of ∼60–70 nt in length by microprocessor complex, which includes RNase III enzyme Drosha and DGCR8 (DiGeorge Syndrome Critical Region Gene 8). Pre-miRs are subsequently exported to the cytoplasm by export 5-Ran-GTP where they are further cleaved by the RNase III enzyme Dicer to generate mature miRs of ∼22 nt in length (11–20). The importance of miRs in regulating cellular functions has been increasingly recognized in several processes including tumorigenesis, tumor invasion and metastasis, cell signaling transduction, stem cell renewal, immune function, apoptosis and reaction to stress (21–25).

The miR-183-96-182 cluster is a critical sensory organ–specific gene that locates to the short arm of chromosome 7 (7q32.2). The cluster is highly expressed in the retina and other sensory organs. Inactivation of the cluster results in early-onset and progressive synaptic defects of the photoreceptors, leading to abnormalities of scotopic and photopic electroretinograms (26). The products of miR-183-96-182 cluster gene, miR-183, miR-96 and miR-182, play important roles in a variety of cancers. For instance, miR-183 promotes cell growth and motility in prostate cancer cells by targeting Dkk-3 and SMAD4 (27). miR-96 promotes hepatocellular carcinoma (HCC) cell proliferation and colony formation by targeting FOXO1 and FOXO3a (28). miR-182 increases tumorigenicity and invasiveness in breast cancer by targeting the matrix metalloproteinase inhibitor RECK (29). The expression levels of the miR-183 family are upregulated in most cancer types (30). But the expression levels of miR-183 family in gastric cancer are controversial. Kong *et al.* (31) found that miR-182 was significantly downregulated in human gastric adenocarcinoma tissue samples. Li *et al.* (32) reported that miR-96, miR-182 and miR-183 were all upregulated in intestinal-type gastric cancers.

Previous reports have demonstrated the interaction between GSK3β and miRs in various human cancers. For instances, GSK3β increases miR-122 level through activating C/EBPα in HCC (33). Inhibition of GSK3β activates miR-181 expression through Wnt/beta-catenin signaling in HCC (34). MiR-26a promotes cholangiocarcinoma via reducing GSK3β expression, resulting in β-Catenin activation (35). The influence and mechanisms of GSK3β on miR biogenesis and function in gastric cancer remain unknown. Here we report that inhibition of GSK3β increases nuclear translocation of β-Catenin, which forms a complex with TCF/LEF-1 to enhance miR-183-96-182 cluster gene expression in gastric cancer cells. Our work identifies miR-183-96-182 cluster gene as a downstream target regulated by β-Catenin/TCF/LEF-1 pathway in gastric cancer cells.

## MATERIALS AND METHODS

### Cell culture and transfection

Wild-type (WT) and GSK3β knockout (KO) mouse embryonic fibroblast (MEF) cells (generous gift from Dr James R. Woodgett) were cultured in Dulbecco’s modified Eagle’s medium (Invitrogen, Carlsbad, CA, USA) with 10% fetal bovine serum (FBS; Thermo Scientific), 2 mM l-glutamine and nonessential amino acids (Invitrogen). AGS cells (ATCC) were cultured in Ham’s F-12 medium (ATCC) plus 10% FBS (Invitrogen). HeLa cells (ATCC) were grown in Eagle’s Minimum Essential Medium (Lonza) supplemented with 10% FBS, 2 mM l-glutamine and nonessential amino acids (Lonza). Cells were trypsinized and reseeded in culture plates 1 day before transfection. AGS cells were transfected with GenJet Plus DNA Transfection Reagent (SignaGen Laboratories) when cell confluency was ∼70%.

### Primary antibodies and primers

GSK3β (3D10) mouse mAb, Lef-1 (C12A5) rabbit mAb, β-Catenin (6B3) rabbit mAb, CK1ε polyclonal antibody, CK2α polyclonal antibody, FoxO1 rabbit mAb and β-Catenin (L87A12) mouse mAb were purchased from Cell Signaling Technology. GAPDH (0411) mouse monoclonal antibody, GAPDH (FL-335) rabbit polyclonal antibody, Lamin A/C (636) mouse mAb and β-actin (R-22) rabbit polyclonal antibody were purchased from Santa Cruz Biotechnology. All primers for mature miRNA detection were purchased from Applied Biosystems; all other primers were ordered from Integrated DNA Technologies. The sequences of the primers are listed in Supplementary Table S1.

### MiRNA array

**Figure 1.**
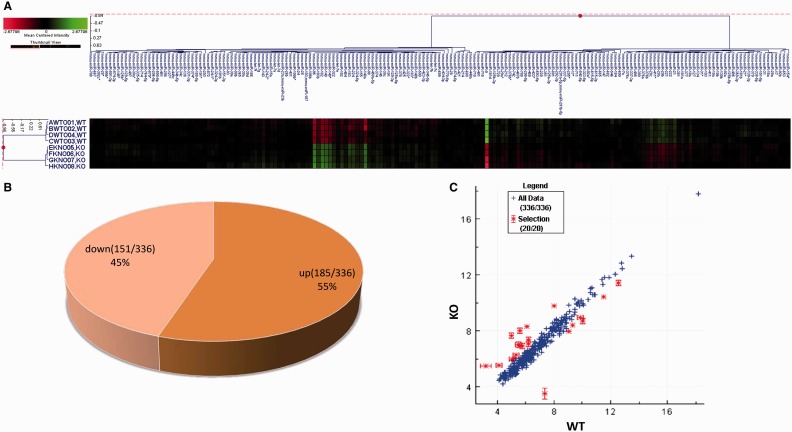
KO of GSK3β changes miRNA expression differentially. Total RNA was extracted from WT or GSK3β KO MEF cells. Four high-quality RNA samples for WT or KO were used for miR array analysis. (A) Agglomerative hierarchical clustering of the processed miR array data using cosine correlation distance metric. (**B**) Percentage of upregulated or downregulated miRs of the 336 measured miRs. (**C**) The top 20 hits have been highlighted on the scatterplot with all 336 miR data points.

Total RNA was extracted from WT and KO MEF cells using TRIZOL (Invitrogen). MiR expression profiling of both WT and KO cells (four replicates each) was performed using a commercial array (Dharmacon Inc of Thermo Scientific). Relative Intensity data for eight samples was subjected to statistical filtering, keeping miR probes with *P* ≤ 0.05 in at least three of the eight experiments. This resulted in 336 miR probes passing statistical filters. The remaining data were inter-array scaled and transformed to log2. The experiments were annotated with factor labels as indicated in Figure 1A. This annotated, filtered, scaled and log2 transformed data set was used for agglomerative hierarchical clustering using cosine correlation distance metric.

### Cytoplasmic and nuclear fractionation

Cytoplasmic and nuclear fractionation was performed using EZ Nuclei Isolation Kit (Sigma) according to the manufacturer’s instructions. Briefly, cells were harvested and washed once with cold phosphate buffered saline. Cells were then suspended in EZ Nuclei Isolation buffer and rotated at 4°C for 5 min. After centrifugation at 4°C for 5 min, supernatant was collected containing the cytoplasmic fraction. Cell lysis and centrifugation were repeated three times. The final pellets were collected as the nuclear fraction and lysed in Pierce IP lysis buffer.

### Western blotting

Gastric cancer samples and the matched control gastric tissues were from Rhode Island Hospital Tissue Bank and their use was approved by Rhode Island Hospital institutional review board (IRB). MEF cell, AGS cell or gastric tissue lysates were prepared in Pierce IP lysis buffer, separated by 4–12% NuPAGE® Novex® 4–12% Bis–Tris gel electrophoresis and electroblotted to nitrocellulose membrane (Bio-Rad). Blotted membranes were probed with their respective primary antibodies, rotating at 4°C overnight. Membranes were washed three times in Tris-Buffered Saline with Tween 20 (TBST) buffer and probed with secondary antibody (Alexa Fluor 680 goat anti-rabbit IgG or IRDye800-conjugated Affinity Purified Anti-Mouse IgG, respectively) at room temperature for 1 h. Membranes were then washed three times in TBST buffer and direct infrared fluorescence detection was performed with a Licor Odyssey® Infrared Imaging System (36). The integrated intensities (counts-mm^2^) of protein bands were quantified according to manufacturer’s instructions. The relative protein level was normalized with the integrated intensity of respective GAPDH.

### Immunohistochemistry

Using the same gastric cancer samples and their matched controls, immunohistochemistry (IHC) was performed on paraffin-embedded tissues sectioned at four microns. Sections were deparaffinized and rehydrated through graded alcohols to distilled water and treated with heat and pressure induced antigen retrieval in 10 mM citrate buffer (pH 6.0) for 10 min and blocked using Peroxidase Block (Dako, Carpinteria, CA) for 5 min. Sections were incubated with GSK3β Rabbit mAb (Cell Signaling) or β-Catenin Rabbit mAb (Cell Signaling) overnight at 4°C. The Envision+Dual Link System Peroxidase (Dako) was used as the secondary antibody followed by Liquid DAB+Substrate ChromogenSystem (Dako). Counterstaining was performed with hematoxylin. The slides were dehydrated and cleared through xylene then coverslipped.

### Real-time reverse transcriptase-polymerase chain reaction

Total RNA was extracted by TRIZOL (Invitrogen) and 1 µg of total RNA was used for cDNA synthesis using MMLV reverse transcriptase (New England Biolabs) as described in the manufacturer’s manual. TaqMan real-time reverse transcriptase-polymerase chain reaction (RT-PCR) miRNA detection kits (Applied Biosystems) that include RT primers and TaqMan probes were used to quantify the levels of mature miRNAs, and 18 S RNA was used for normalization. All PCR reactions were run in triplicate.

### Luciferase assay

A DNA fragment of 2340 base pairs from the upstream region of the miR-183-96-182 cluster containing the putative TCF/LEF-1 binding elements (TBEs) was amplified from the genomic DNA of AGS cells and subcloned into the pSwitchlight_Prom Promoter Reporter Vector (SwitchGear Genomics) between SacI and HindIII sites (sense primer: ACCTGAGCTCTCTCGACTTTC; antisense primer: AGTTAAGCTTCCTGCGCCGG). The newly cloned construct was named pmiR-96 cluster promoter. AGS cells were transfected with pmiR-96 cluster promoter plus indicated constructs or the empty reporter. A β-Gal plasmid was cotransfected with the reporter constructs, respectively, to control for transfection efficiency. Twenty-four hours after transfection, the cells were harvested for luciferase assay. Renilla luciferase activities were quantified using LightSwitch Luciferase Assay Reagent LS010 (SwitchGear Genomics), and Renilla luciferase activity was normalized to β-Gal activity. For each experiment, a control using an empty vector (EV) was used and corrected luciferase values were averaged, arbitrarily set to a value of ‘1’ and served as a reference for comparison of fold differences in experimental values.

### Chromatin immunoprecipitation assay

Chromatin immunoprecipitation (ChIP) assays were performed using a SimpleChIP® Enzymatic Chromatin IP Kit (Magnetic Beads) from Cell Signal Technology following the manufacturer’s protocol. Briefly, AGS or Hela cells were fixed with 1% formaldehyde for 10 min to cross-link proteins to DNA. Nuclei were prepared and treated with Micrococcal Nuclease for 20 min at 37°C to digest the chromatin into 150–900 bp DNA/protein fragments. β-Catenin rabbit mAb and ChIP Grade Protein G Magnetic Beads were used to immunoprecipitate β-Catenin/TCF/LEF-1 bound DNA fragments. Normal Rabbit IgG was used as a negative control. After chromatin was eluted from the beads, the cross-links were reversed by adding NaCl and Proteinase K and incubating for 2 h at 65°C. DNA was purified with spin column and used for standard PCR and quantitative real-time PCR. We used Native Pfu DNA Polymerase (Stratagene) for standard PCR and RT^2^ Real-Time™ SYBR Green PCR Master Mix (Thermo Fisher/Fermentas) for quantitative real-time PCR according to the manufacturer’s instructions.

### Cell Proliferation and migration assays

To suppress the miR-183-96-182 cluster, AGS cells were transfected with miRCURY LNA™ Inhibitors (Exiqon). Their respective sequences are: miRCURY LNA™ miRNA Inhibitor Negative Control A: GTGTAACACGTCTATACGCCCA; miRCURY LNA™ miR-183 inhibitor: AGTGAATTCTACCAGTGCCAT; miRCURY LNA™ miR-96 inhibitor: GCAAAAATGTGCTAGTGCCAA; miRCURY LNA™ miR-182 inhibitor: TGTGAGTTCTACCATTGCCAA. To knock down GSK3β, AGS cells were transfected with GSK3B Pre-design Chimera RNAi or negative control Naito 1 Pre-design Chimera RNAi (Abnova). Forty-eight hours after transfection, the cells were trypsinized and cultured for another 24 h in either 96-well flat-bottom plate for cell proliferation assay, in Boyden Chamber 12-well Cell Culture Insert (BD Falcon™) for migration assay, or in 12-well plate for western blot. A cell proliferation assay was performed with a colorimetric WST-1 assay kit (Roche Applied Science) according to the manufacturer’s instructions. In the Boyden Chamber migration assay, cells migrated from the upper chamber (5% FBS) to the lower one (10% FBS) were collected and counted. We set the control as ‘1’ arbitrarily to quantify the proliferation or migration of the cells.

### Statistical analysis

Quantitative data were analyzed by unpaired Student’s *t*-test. The miR array data were analyzed by textbook analysis of variance (ANOVA), with FDR multiple test correction, across the ‘Group’ factor (KO versus WT). The raw ANOVA results are reported in the form of agglomerative hierarchical clustering graphic.

## RESULTS

### KO of GSK3β changes miR expression differentially

The raw ANOVA miR array results are reported in the form of agglomerative hierarchical clustering graphic (Figure 1A). Of the 336 measured miRs, 55% (185 of 336) were upregulated and 45% (78 of 336) downregulated (Figure 1B). The top 20 differentially expressed miRs by fold change are listed in the Table 1, where the direction of change is relative to factor level WT. These hits have been highlighted on the scatter plot with all 336 miR data points (Figure 1C).

**Table 1.** The top 20 differentially expressed miRs by fold change

Sequence code	Intensity (KO)	Intensity (WT)	Fold change	Direction
mmu-miR-9	3.46168	7.36237	14.93566	DOWN
mmu-miR-96	7.62672	5.01815	6.09897	UP
mmu-miR-182	7.96993	5.62138	5.09311	UP
mmu-miR-148a	5.41639	3.2136	4.60371	UP
mmu-miR-140	8.25698	6.11195	4.423	UP
mmu-miR-140*	9.74879	8.01526	3.32539	UP
mmu-miR-183	6.96582	5.51917	2.72575	UP
mmu-miR-29b	8.65609	10.03812	2.60634	DOWN
mmu-miR-224	5.47956	4.15714	2.50084	UP
mmu-miR-193b	6.87893	5.63272	2.37217	UP
mmu-miR-21	11.34134	12.51489	2.25566	DOWN
mmu-miR-29c	7.93012	9.06697	2.199	DOWN
mmu-miR-29a	10.40129	11.52748	2.18281	DOWN
mmu-miR-152	6.88774	5.77899	2.15658	UP
mmu-miR-322	7.32264	6.22746	2.13641	UP
mmu-miR-221	8.35923	9.33936	1.97265	DOWN
mmu-miR-487b	8.90009	9.84554	1.92579	DOWN
mmu-miR-155	6.23521	5.32532	1.87891	UP
mmu-miR-324-5p	5.95074	5.07725	1.83209	UP
mmu-miR-374	7.02733	6.23325	1.73397	UP

### KO of GSK3β increases protein level and nuclear translocation of β-Catenin

**Figure 2.**
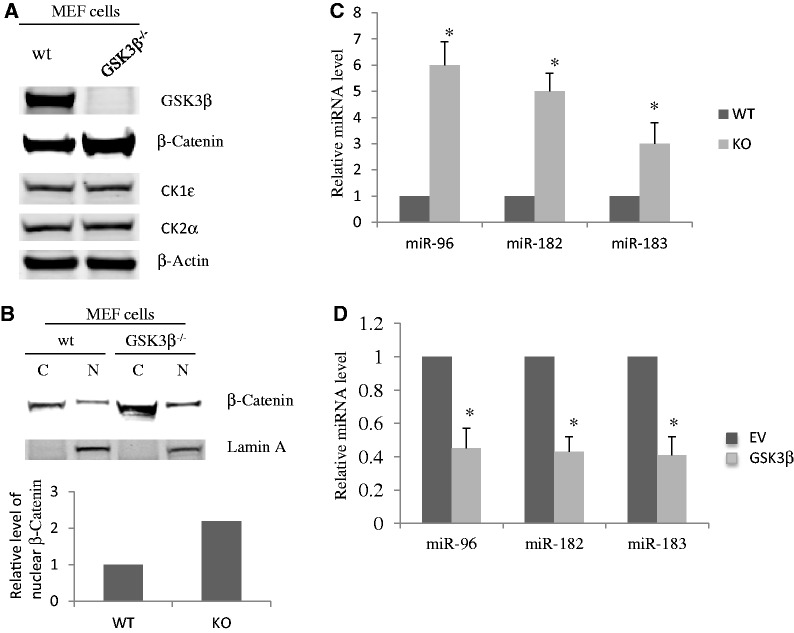
KO of GSK3β increases protein level and nuclear translocation of β-Catenin. (**A**) GSK3β KO increased β-Catenin expression level. Whole-cell lysates were prepared from WT or GSK3β KO MEF cells, respectively, and protein levels of GSK3β, β-Catenin, CK1ε, CK2α and β-Actin were resolved by western blotting (WB). (**B**) β-Catenin protein translocates into the nucleus in GSK3β KO MEF cells. Cytoplasmic and nuclear fractions were prepared from WT or KO MEF cells, respectively, and β-Catenin protein levels were determined by WB. (**C**) MiR array analysis showed that GSK3β KO increased the expression of miR-96, miR-182 and miR-183 6-, 5- or 3-fold, respectively. (*P < 0.05, by Student’s *t*-test). (**D**) Increase of GSK3β protein level inhibited the expression of miR-96, miR-182 and miR-183 in AGS cells. A construct encoding GSK3β was transfected into AGS cells. Forty-eight hours after transfection, total RNA was extracted and used for RT-PCR. All experiments were repeated three times with similar results (*P < 0.05 by Student’s *t*-test).

GSK3β phosphorylates β-Catenin that is primed by other kinases such as casein kinases 1 and 2, a necessary prerequisite to its entry into the ubiquitin-proteasome pathway for degradation (5). We first quantified protein levels of β-Catenin, GSK3β, CK1ε and CK2α in WT and GSK3β KO MEF cells. As expected, GSK3β KO increased β-Catenin expression level by 2-fold but had no effects on CK1 and CK2 expression (Figure 2A). To determine if β-Catenin protein translocation into the nucleus was increased in GSK3β KO MEF cells, we fractionated the cytoplasmic and nuclear parts of MEF cells and found, as expected, that the nuclear β-Catenin protein levels were also increased by 2-fold in GSK3β KO MEF cells (Figure 2B). Our previous studies have shown that phosphorylation of Drosha by GSK3β facilitates its nuclear localization (9,10). Unexpectedly, GSK3β KO also increased some miR expression. Of the miRs that were increased the most by GSK3β KO, miR-96, miR-182 and miR-183 are all from the same miR gene cluster. The miR array data revealed that they were increased 6-, 5- or 3-fold, respectively (Table 1 and Figure 2C), suggesting that GSK3β may suppress the generation of miR-96, miR-182 and miR-183. To further verify this, we ectopically expressed a GSK3β construct in human gastric epithelial AGS cells. Compared with EV, overexpression of GSK3β inhibited the expression of miR-96, miR-182 and miR-183 by 2-fold (*P* < 0.05) (Figure 2D).

### Expression levels of GSK3β, β-Catenin, miR-96, miR-182, miR-183 and primary miR-183-96-182 cluster in human gastric cancer

**Figure 3.**
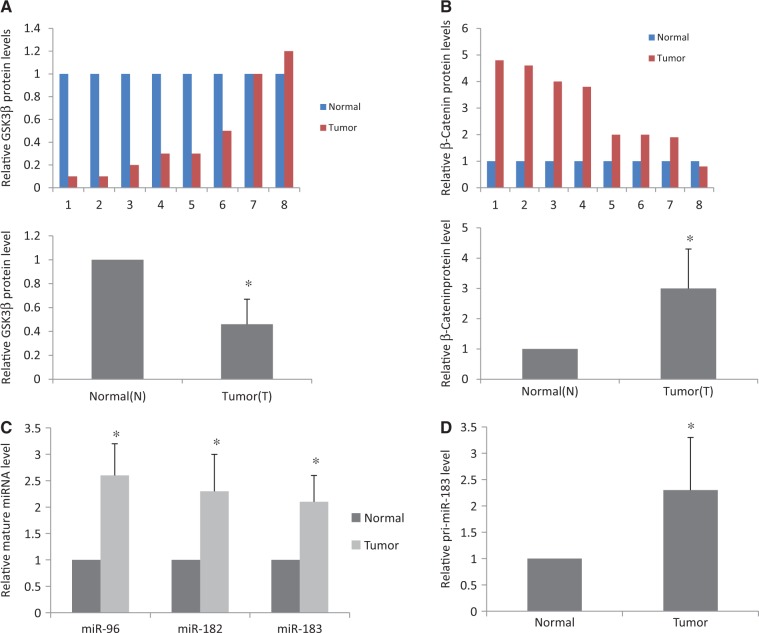
Expression levels of GSK3β, β-Catenin, miR-96, miR-182, miR-183 and pri-miR-183 in human gastric cancer. (**A**) GSK3β protein levels in eight human gastric cancer tissues and matched normal tissues determined by WB. The integrated intensity (counts-mm^2^) of each GSK3β band was quantified and normalized with that of respective GAPDH. The upper panel shows individual quantifications. Statistical analysis of the normalized density is shown in bottom panel. GSK3β protein level decreased 2-fold in gastric cancer (*n* = 8, *P < 0.05 by Student’s *t*-test). (**B**) β-Catenin protein levels in eight human gastric cancer tissues and matched normal tissues determined by WB. The integrated intensity (counts-mm^2^) of each β-Catenin band was normalized with that of respective GAPDH. The upper panel shows individual quantifications. Statistical analysis of the normalized density is shown in bottom panel. β-Catenin protein level increased 3-fold in gastric cancer (*n* = 8, *P < 0.05 by Student’s *t*-test). (**C**) The expression levels of miR-96, miR-182 and miR-183 were increased in gastric cancer samples compared with the matched normal tissues. Total RNA was extracted using TRIZOL and miRs were measured by means of TaqMan real-time RT-PCR miR detection kits. (**D**) The pri-miR-183 level in gastric cancer samples and in the matched normal tissues. Total RNA from the tumor and matched normal tissues was used for RT-PCR to measure pri-miR-183 level. All RT-PCR experiments were performed in triplicate (*n* = 8, *P < 0.05 by Student’s *t*-test).

**Figure 4.**
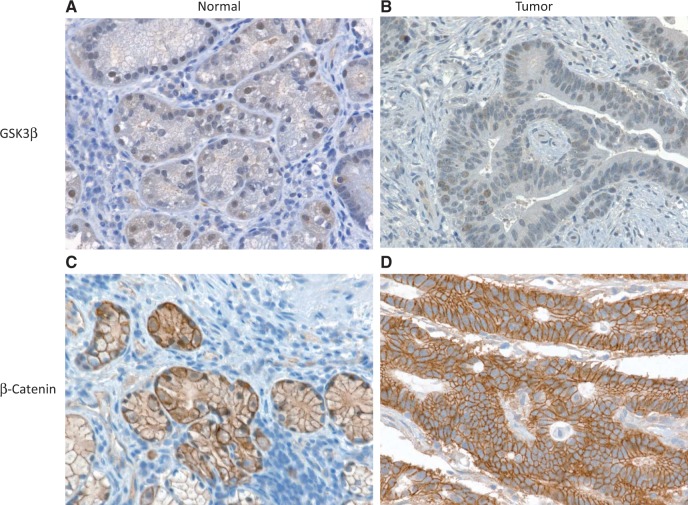
Confirmation of the expression of GSK3β and β-Catenin by IHC. Eight pairs of gastric cancer and adjacent normal tissue samples from eight different patients were used for IHC. The IHC slides were blindly analyzed by pathologists, and representative images were taken by an imaging specialist. (**A**) GSKβ expression in matched normal control gastric tissue. (**B**) GSKβ expression in gastric cancer tissue. (**C**) β-Catenin expression in matched normal control gastric tissue. (**D**) β-Catenin expression in gastric cancer tissue from the same subject. GSKβ expression in gastric cancer (B) was lower than in surrounding normal tissue (A). β-Catenin expression in gastric cancer (D) was higher than in surrounding normal tissue (C).

Since GSK3β inhibits the expression of miR-96, miR-182 and miR-183 in human gastric epithelial AGS cells, we measured the protein levels of GSK3β and β-Catenin by western blot and miR levels of miR-96, miR-182 and miR-183 by quantitative RT-PCR (qRT-PCR) in eight gastric cancer and matched normal gastric tissue samples. As shown in Figure 3A, the overall GSK3β protein level in gastric cancer samples was ∼50% of that in the matched normal samples (*n* = 8, *P* < 0.05). β-Catenin levels were increased ∼2-fold in gastric cancer samples compared with matched normal gastric tissue samples (Figure 3B). We further confirmed the changes of the expression levels of GSK3β and β-Catenin by IHC (Figure 4). The levels of miR-96, miR-182 and miR-183 in gastric cancer were increased by 2-fold (Figure 3C). Surprisingly, the primary miR-183-96-182 cluster (pri-miR-183) levels were higher in gastric cancer tissues than that in the matched normal tissues, indicating that GSK3β regulates the production of miR-96, miR-182 and miR-183 through β-Catenin at the transcription level.

### β-Catenin/TCF/LEF-1 binds to and activates the promoter of miR-183-96-182 cluster gene

**Figure 5.**
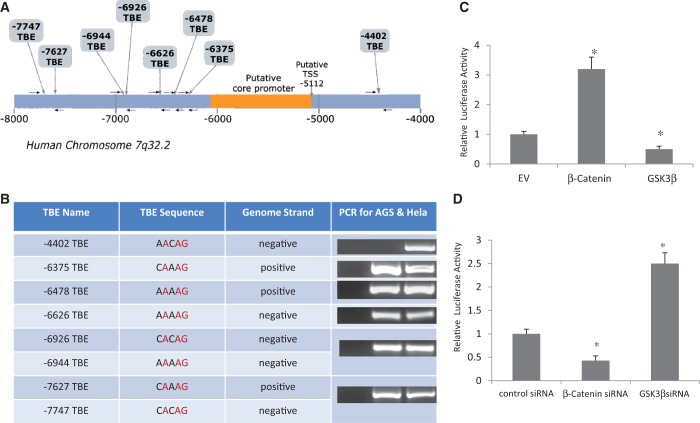
β-Catenin/TCF/Lef-1 binds to and activates the promoter of miR-183-96-182 cluster gene. (**A**) Schematic illustration of the promoter region of the miR-183-96-182 cluster gene showing the locations of the core promoter and putative TBEs. The first nucleotide of miR-96 was set as 1. (**B**) ChIP assay experiments were performed using a SimpleChIP® Enzymatic Chromatin IP Kit and a rabbit mAb against β-Catenin. Five binding sites for β-Catenin/TCF/Lef-1 complex were confirmed in AGS cells. An extra site downstream of the putative core promoter was confirmed in HeLa cells. (**C**) A renilla luciferase construct was generated by subcloning the upstream TBEs containing a DNA fragment into a luciferase vector. Cotransfection of a construct encoding β-Catenin together with the luciferase vector into AGS cells increased the renilla luciferase activity while cotransfection of a construct encoding GSK3β had the opposite effect (compared with EV, *P < 0.05 by Student’s *t*-test). (**D**) Knockdown of β-Catenin significantly decreased renilla luciferase activity, while knockdown of GSK3β increased renilla luciferase activity. The same luciferase construct as in Figure 4C was cotransfected with β-Catenin siRNA or GSK3β siRNA, respectively, into AGS cells (compared with control siRNA, *P < 0.05 by Student’s *t*-test). All experiments were repeated three times with similar results.

The gene encoding miR-96, miR-182 and miR-183 locates to human chromosome 7q32.2. *In silico* screening identified seven potential TBEs in the promoter region of miR-96-182-183 cluster gene (Figure 5A). To determine if these TBEs are bona fide binding sites for β-Catenin/TCF/LEF-1 complex, we performed ChIP experiments using a SimpleChIP® Enzymatic Chromatin IP Kit and a rabbit mAb against β-Catenin. We confirmed that all the TBEs upstream of the putative core promoter were bona fide binding sites for β-Catenin/TCF/LEF-1 complex in AGS cells (Figure 5B). In HeLa cells, we also confirmed another TBE downstream of the core promoter (Figure 5B). To determine if the binding of β-Catenin/TCF/LEF-1 complex to TBEs is functional, we generated a renilla luciferase construct by subcloning the upstream TBEs containing DNA fragment into a luciferase vector. Cotransfection of a construct encoding β-Catenin together with the luciferase vector in AGS cells increased the renilla luciferase activity by 3-fold (compared with EV, *P* < 0.05), while cotransfection of a construct encoding GSK3β had the opposite effect (2-fold reduction; compared with EV, *P* < 0.05) (Figure 5C). To further confirm the effect of β-Catenin and GSK3β on promoter function, we knocked down β-Catenin or GSK3β with respective specific siRNA molecules. Knockdown of β-Catenin decreased renilla luciferase activity by 2-fold (compared with control siRNA, *P* < 0.05), while knockdown of GSK3β increased renilla luciferase activity by 2.5-fold (compared with control siRNA, *P* < 0.05) (Figure 5D).

### β-Catenin enhances expression of primary and mature miR-96, miR-182 and miR-183

**Figure 6.**
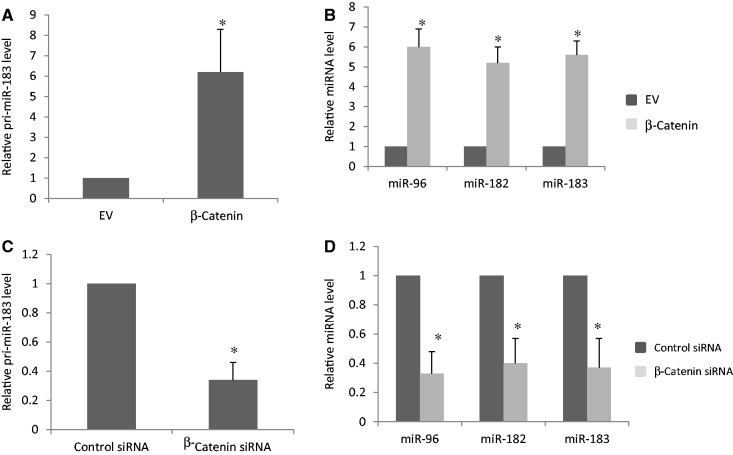
β-Catenin enhances expression of primary and mature miR-96, miR-182 and miR-183. An EV, a vector encoding β-Catenin, control siRNA or β-Catenin siRNA, was transfected into AGS cells, respectively. Total RNA was extracted and used for RT-PCR to measure the expression levels of primary and mature miRs. All experiments were repeated three times with similar results (*P < 0.05 by Student’s *t*-test). (**A**) Overexpression of β-Catenin increases the pri-miR-183 level. (**B**) Overexpression of β-Catenin increases the expression of miR-96, miR-182 and miR-183. (**C**) Knockdown of β-Catenin decreases the pri-miR-183 level. (**D**) Knockdown of β-Catenin decreases the expression of miR-96, miR-182 and miR-183.

To further confirm whether β-Catenin modulates the generation of miR-96, miR-182 and miR-183, we transfected a construct encoding β-Catenin into AGS cells and measured the primary and mature miR levels of miR-96, miR-182 and miR-183. Overexpression of β-Catenin increased the levels of primary and mature miR-96, miR-182 and miR-183 by 5-fold (Figure 6A and B). On the other hand, knockdown of β-Catenin by specific siRNA decreased the primary and mature miR-96, miR-182 and miR-183 levels by 3-fold (Figure 6C and D).

### Suppression of miR-183-96-182 cluster or knockdown of GSK3β alters gastric cancer cell phenotype

**Figure 7.**
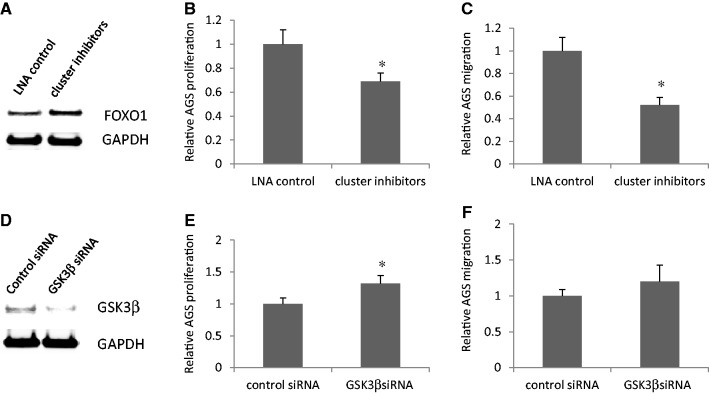
Suppression of miR-183-96-182 cluster or knockdown of GSK3β alters gastric cancer cell phenotype. (**A**) Suppression of miR-183-96-182 cluster increases FoxO1 protein level. (**B**) Suppression of miR-183-96-182 cluster decreases AGS cell proliferation. (**C**) Suppression of miR-183-96-182 cluster decreases AGS cell migration. (**D**) GSK3β siRNA specifically downregulates GSK3β protein. (**E**) Knockdown of GSK3β increases AGS cell proliferation. (**F**) Knockdown of GSK3β does not affect AGS cell migration significantly. All experiments were repeated three times with similar results (*P < 0.05 by Student’s *t*-test).

To investigate the effects of suppression of miR-183-96-182 cluster on gastric cancer cell phenotype, we transfected a miRCURY LNA™ miRNA Inhibitor Negative Control or a mix of miRCURY LNA™ inhibitors for miR-183, miR-96 and miR-182 into AGS cells. The upregulation of FoxO1, one of the targets of miR-183-96-182 cluster, indicated the efficiency of miR inhibitors (Figure 7A). Suppression of miR-183-96-182 cluster decreased proliferation and migration of AGS cells (Figure 7B and C). To investigate the effects of GSK3β knockdown on gastric cancer phenotype, we transfected control siRNA or GSK3β-specific siRNA into AGS cells. Compared with control siRNA, GSK3β siRNA specifically downregulated GSK3β protein (Figure 7D). Knockdown of GSK3β increased AGS cell proliferation (Figure 7E), but had no significant effect on AGS cell migration (Figure 7F).

## DISCUSSION

The Wnt signaling plays a pivotal role in tumorigenesis in various cancers including gastric cancer (37,38). Given that the CK1 and CK2 protein kinase families play important roles in Wnt signaling pathway (39,40), we wondered whether KO GSK3β deregulated the expression of these kinases. We found, however, that knocking out GSK3β did not change the expression of CK1 and CK2, ruling out deregulated activity of these kinases in GSK3β KO cells. As a key component of this pathway, GSK3β has emerged as a potential therapeutic target for cancer treatment (41). Because GSK3β is a multifunctional protein kinase, inhibition of GSK3β may have serious side effects. To reduce these side effects, miR-183-96-182 cluster could serve as a potential downstream target of the Wnt signaling pathway for treatment of gastric cancer and deserves further exploration.

β-Catenin/TCF/LEF-1 complex binds to a region near the core promoter of the miR-183-96-182 cluster gene. Various other transcription factors bind to this region as well, indicating that the cluster gene is potentially regulated by many other transcription factors in addition to TCF and LEF-1 (Supplementary Figure S1).

We measured pri-miR-183 and mature miR-96, miR-182, miR-183 expression levels in gastric cancer and matched normal gastric tissue by qRT-PCR. Our results showed that both the primary and mature miR-96, miR-182, miR-183 expression levels were significantly upregulated in gastric cancer tissues compared with the adjacent normal control gastric tissues. By means of western blotting and IHC techniques, we found that GSK3β protein expression decreased and β-Catenin protein level increased significantly in gastric cancer. We hypothesized that GSK3β regulates miR-183-96-182 cluster through β-Catenin/TCF/LEF-1 pathway in gastric cancer cells. Using miR array, ChIP assay, luciferase assay, qRT-PCR, we confirmed our hypothesis and identified miR-183-96-182 cluster as a novel target of the β-Catenin/TCF/LEF-1 pathway in gastric cancer cells.

Gastric cancer, the fourth most common cancer and the second leading cause of cancer-related deaths in the world, is one of the major threats to human health. According to the World Health Organization, gastric cancer annually claims ∼800 000 lives worldwide, metastatic disease being uniformly fatal (42). In this study, we found that miR-183-96-182 cluster inhibitors decrease the proliferation and migration of gastric cancer AGS cells and provide a functional link between GSK3β, the miRNA-183-96-182 cluster and the β-Catenin/TCF/LEF-1 pathway in gastric cancer.

## SUPPLEMENTARY DATA

Supplementary Data are available at NAR Online.

## FUNDING

National Institutes of Health (NIH) [P20GM103421, P20GM103468 to B.R.]; Lifespan/Brown/Tufts CFAR [P30AI042853 to B.R.]; National Institutes of Health [T32DA013911 to X.T.]; National Natural Scientific Foundation of China [81172296 to X.T.]. Funding for open access charge: NIH.

*Conflict of interest statement*. None declared.

## Supplementary Material

Supplementary Data

## References

[1] Hoeflich KP, Luo J, Rubie EA, Tsao MS, Jin O, Woodgett JR (2000). Requirement for glycogen synthase kinase-3beta in cell survival and NF-kappaB activation. Nature.

[2] Rubinfeld B, Albert I, Porfiri E, Fiol C, Munemitsu S, Polakis P (1996). Binding of GSK3beta to the APC-beta-catenin complex and regulation of complex assembly. Science.

[3] Behrens J, von Kries JP, Kühl M, Bruhn L, Wedlich D, Grosschedl R, Birchmeier W (1996). Functional interaction of beta-catenin with the transcription factor LEF-1. Nature.

[4] Behrens J, Jerchow BA, Würtele M, Grimm J, Asbrand C, Wirtz R, Kühl M, Wedlich D, Birchmeier W (1998). Functional interaction of an axin homolog, conductin, with beta-catenin, APC, and GSK3beta. Science.

[5] Aberle H, Bauer A, Stappert J, Kispert A, Kemler R (1997). beta-catenin is a target for the ubiquitin-proteasome pathway. EMBO J..

[6] He TC, Sparks AB, Rago C, Hermeking H, Zawel L, da Costa LT, Morin PJ, Vogelstein B, Kinzler KW (1998). Identification of c-MYC as a target of the APC pathway. Science.

[7] Mann B, Gelos M, Siedow A, Hanski ML, Gratchev A, Ilyas M, Bodmer WF, Moyer MP, Riecken EO, Buhr HJ (1999). Target genes of beta-catenin-T cell-factor/lymphoid-enhancer-factor signaling in human colorectal carcinomas. Proc. Natl Acad. Sci. USA.

[8] Tetsu O, McCormick F (1999). Beta-catenin regulates expression of cyclin D1 in colon carcinoma cells. Nature.

[9] Tang X, Zhang Y, Tucker L, Ramratnam B (2010). Phosphorylation of the RNase III enzyme Drosha at Serine300 or Serine302 is required for its nuclear localization. Nucleic Acids Res..

[10] Tang X, Li M, Tucker L, Ramratnam B (2011). Glycogen Synthase Kinase 3 beta (GSK3β) phosphorylates the RNAase III enzyme Drosha at S300 and S302. PLoS One.

[11] Lee RC, Feinbaum RL, Ambros V (1993). The *C*. *elegans* heterochronic gene lin-4 encodes small RNAs with antisense complementarity to lin-14. Cell.

[12] Han J, Lee Y, Yeom KH, Kim YK, Jin H, Kim VN (2004). The Drosha-DGCR8 complex in primary microRNA processing. Genes Dev..

[13] Lee Y, Ahn C, Han J, Choi H, Kim J, Yim J, Lee J, Provost P, Ra dmark O, Kim S (2003). The nuclear RNase III Drosha initiates microRNA processing. Nature.

[14] Han J, Lee Y, Yeom KH, Nam JW, Heo I, Rhee JK, Sohn SY, Cho Y, Zhang BT, Kim VN (2006). Molecular basis for the recognition of primary microRNAs by the.complex. Cell.

[15] Lee Y, Kim M, Han J, Yeom KH, Lee S, Baek SH, Kim VN (2004). MicroRNA genes are transcribed by RNA polymerase II. EMBO J..

[16] Borchert GM, Lanier W, Davidson BL (2006). RNA polymerase III transcribes human microRNAs. Nat. Struct. Mol. Biol..

[17] Landthaler M, Yalcin A, Tuschl T (2004). The human DiGeorge syndrome critical region gene 8 and its *D*. *melanogaster* homolog are required for miRNA biogenesis. Curr. Biol..

[18] Lee Y, Han J, Yeom KH, Jin H, Kim VN (2006). Drosha in primary microRNA processing. Cold Spring Harb. Symp. Quant. Biol..

[19] Winter J, Jung S, Keller S, Gregory RI, Diederichs S (2009). Many roads to maturity: microRNA biogenesis pathways and their regulation. Nat. Cell Biol..

[20] Kim VN, Han J, Siomi MC (2009). Biogenesis of small RNAs in animals. Nat. Rev. Mol. Cell Biol..

[21] Gregory RI, Shiekhattar R (2005). MicroRNA biogenesis and cancer. Cancer Res..

[22] Ma L, Teruya-Feldstein J, Weinberg RA (2007). Tumour invasion and metastasis initiated by microRNA-10b in breast cancer. Nature.

[23] Zhang W, Dahlberg JE, Tam W (2007). MicroRNAs in tumorigenesis: a primer. Am. J. Pathol..

[24] John B, Enright AJ, Aravin A, Tuschl T, Sander C, Marks DS (2004). Human microRNA targets. PLoS Biol..

[25] Leung AK, Sharp PA (2007). microRNAs: a safeguard against turmoil?. Cell.

[26] Lumayag S, Haldin CE, Corbett NJ, Wahlin KJ, Cowan C, Turturro S, Larsen PE, Kovacs B, Witmer PD, Valle D (2013). Inactivation of the microRNA-183/96/182 cluster results in syndromic retinal degeneration. Proc. Natl Acad. Sci. USA.

[27] Ueno K, Hirata H, Shahryari V, Deng G, Tanaka Y, Tabatabai ZL, Hinoda Y, Dahiya R (2013). microRNA-183 is an oncogene targeting Dkk-3 and SMAD4 in prostate cancer. Br. J. Cancer.

[28] Xu D, He X, Chang Y, Xu C, Jiang X, Sun S, Lin J (2013). Inhibition of miR-96 expression reduces cell proliferation and clonogenicity of HepG2 hepatoma cells. Oncol. Rep..

[29] Chiang CH, Hou MF, Hung WC (2013). Up-regulation of miR-182 by β-catenin in breast cancer increases tumorigenicity and invasiveness by targeting the matrix metalloproteinase inhibitor RECK. Biochim. Biophys. Acta.

[30] Zhang QH, Sun HM, Zheng RZ, Li YC, Zhang Q, Cheng P, Tang ZH, Huang F (2013). Meta-analysis of microRNA-183 family expression in human cancer studies comparing cancer tissues with noncancerous tissues. Gene.

[31] Kong WQ, Bai R, Liu T, Cai CL, Li X, Tang H (2012). MicroRNA-182 targets cAMP-responsive element-binding protein 1 and suppresses cell growth in human gastric adenocarcinoma. FEBS J..

[32] Li X, Luo F, Li Q, Xu M, Feng D, Zhang G, Wu W (2011). Identification of new aberrantly expressed miRNAs in intestinal-type gastric cancer and its clinical significance. Oncol. Rep..

[33] Zeng C, Wang R, Li D, Lin XJ, Wei QK, Yuan Y, Wang Q, Chen W, Zhuang SM (2010). A novel GSK-3 beta-C/EBP alpha-miR-122-insulin-like growth factor 1 receptor regulatory circuitry in human hepatocellular carcinoma. Hepatology.

[34] Ji J, Yamashita T, Wang XW (2011). Wnt/beta-catenin signaling activates microRNA-181 expression in hepatocellular carcinoma. Cell Biosci..

[35] Zhang J, Han C, Wu T (2012). MicroRNA-26a promotes cholangiocarcinoma growth by activating β-catenin. Gastroenterology.

[36] Tang X, Gao JS, Guan YJ, McLane KE, Yuan ZL, Ramratnam B, Chin YE (2007). Acetylation-dependent signal transduction for type I interferon receptor. Cell.

[37] Klaus A, Birchmeier W (2008). Wnt signaling and its impact on development and cancer. Nat. Rev. Cancer.

[38] Radulescu S, Ridgway RA, Cordero J, Athineos D, Salgueiro P, Poulsom R, Neumann J, Jung A, Patel S, Woodgett J (2013). Acute WNT signalling activation perturbs differentiation within the adult stomach and rapidly leads to tumour formation. Oncogene.

[39] Bernatik O, Ganji RS, Dijksterhuis JP, Konik P, Cervenka I, Polonio T, Krejci P, Schulte G, Bryja V (2011). Sequential activation and inactivation of Dishevelled in the Wnt/beta-catenin pathway by casein kinases. J. Biol. Chem..

[40] Bryja V, Schambony A, Cajánek L, Dominguez I, Arenas E, Schulte G (2008). Beta-arrestin and casein kinase 1/2 define distinct branches of non-canonical WNT signalling pathways. EMBO Rep..

[41] Anastas JN, Moon RT (2013). WNT signalling pathways as therapeutic targets in cancer. Nat. Rev. Cancer.

[42] Lozano R, Naghavi M, Foreman K, Lim S, Shibuya K, Abovans V, Abraham J, Adair T, Aggarwal R, Ahn SY (2012). Global and regional mortality from 235 causes of death for 20 age groups in 1990 and 2010: a systematic analysis for the global burden of disease study 2010. Lancet.

