# Supplementary material for: Glycogen synthase kinase 3 beta inhibits microRNA-183-96-182 cluster via the β-Catenin/TCF/LEF-1 pathway in gastric cancer cells
Source: Nucleic Acids Res. 2013 Dec 12;42(5):2988–98. doi: 10.1093/nar/gkt1275 (PMC3950676; doi:10.1093/nar/gkt1275)
Supplement: Supplementary Data [file supp_42_5_2988__index.html]

Glycogen synthase kinase 3 beta inhibits microRNA-183-96-182 cluster via the β-Catenin/TCF/LEF-1 pathway in gastric cancer cells — Glycogen synthase kinase 3 beta inhibits microRNA-183-96-182 cluster via the β-Catenin/TCF/LEF-1 pathway in gastric cancer cells — Supplementary Data 

# Glycogen synthase kinase 3 beta inhibits microRNA-183-96-182 cluster via the β-Catenin/TCF/LEF-1 pathway in gastric cancer cells

## Supplementary Data

files

**Files in this Data Supplement:**

- Supplementary Data - pptx file
- Supplementary Data - docx file
