# Supplementary material for: Glycogen synthase kinase 3 beta inhibits microRNA-183-96-182 cluster via the β-Catenin/TCF/LEF-1 pathway in gastric cancer cells
Source: Nucleic Acids Res. 2013 Dec 12;42(5):2988–98. doi: 10.1093/nar/gkt1275 (PMC3950676; doi:10.1093/nar/gkt1275)
Supplement: Supplementary Data [file supp_gkt1275_nar-02232-y-2013-File009.pptx]

## Slide 1
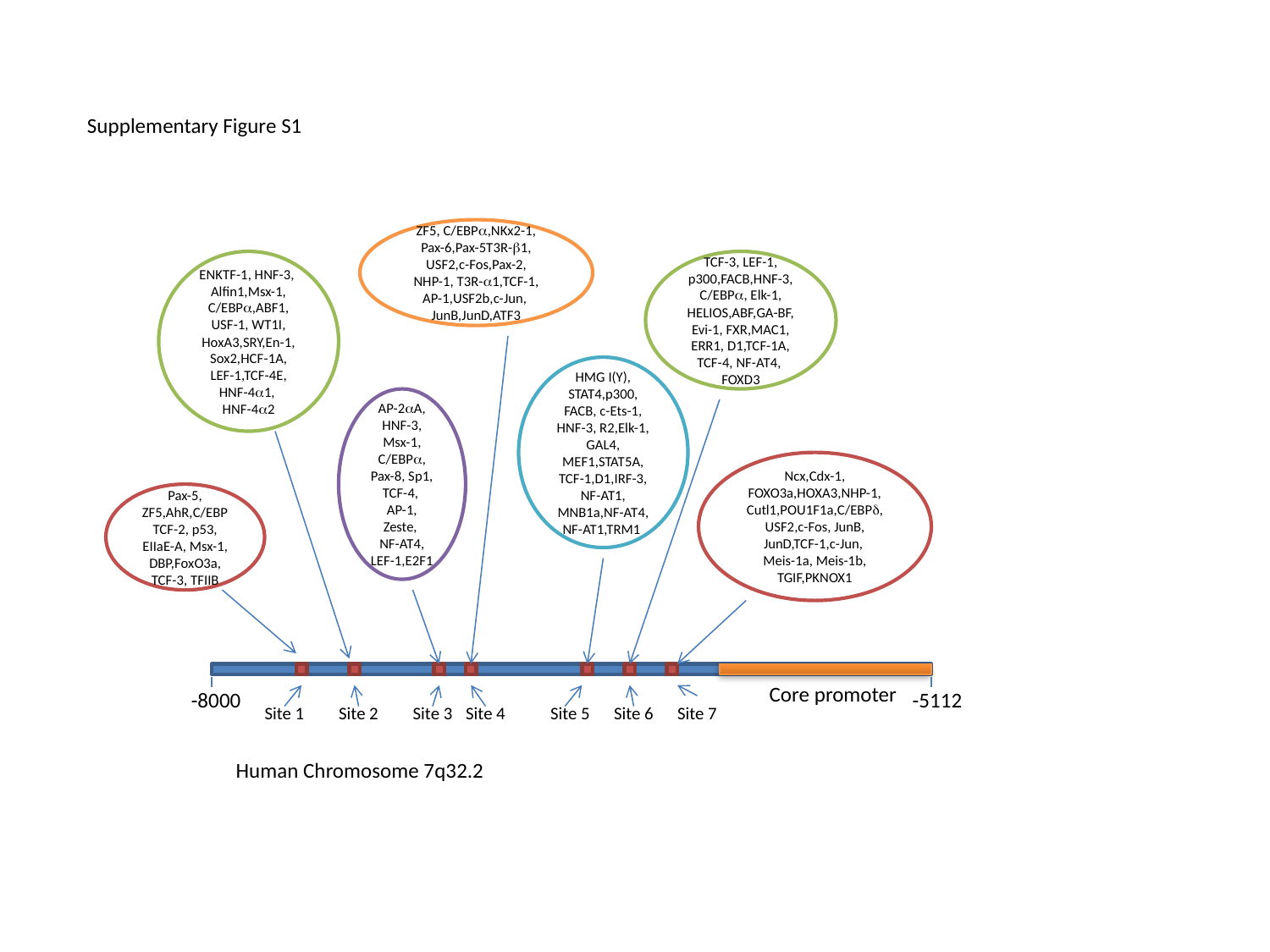

Supplementary Figure S1
ZF5, C/EBPα,NKx2-1, Pax-6,Pax-5T3R-β1, USF2,c-Fos,Pax-2,
NHP-1, T3R-α1,TCF-1, AP-1,USF2b,c-Jun, JunB,JunD,ATF3
ENKTF-1, HNF-3,
Alfin1,Msx-1, C/EBPα,ABF1,
USF-1, WT1I, HoxA3,SRY,En-1, Sox2,HCF-1A, LEF-1,TCF-4E, HNF-4α1,
HNF-4α2
TCF-3, LEF-1, p300,FACB,HNF-3, C/EBPα, Elk-1, HELIOS,ABF,GA-BF, Evi-1, FXR,MAC1, ERR1, D1,TCF-1A, TCF-4, NF-AT4, FOXD3
HMG I(Y), STAT4,p300,
FACB, c-Ets-1, HNF-3, R2,Elk-1, GAL4, MEF1,STAT5A, TCF-1,D1,IRF-3, NF-AT1, MNB1a,NF-AT4, NF-AT1,TRM1
AP-2αA,
HNF-3, Msx-1, C/EBPα, Pax-8, Sp1, TCF-4,
AP-1, Zeste,
NF-AT4, LEF-1,E2F1
Ncx,Cdx-1, FOXO3a,HOXA3,NHP-1, Cutl1,POU1F1a,C/EBPδ,USF2,c-Fos, JunB, JunD,TCF-1,c-Jun,
Meis-1a, Meis-1b, TGIF,PKNOX1
Pax-5, ZF5,AhR,C/EBP
TCF-2, p53, EIIaE-A, Msx-1, DBP,FoxO3a, TCF-3, TFIIB
Core promoter
-8000
-5112
Site 1
Site 2
Site 3
Site 4
Site 5
Site 6
Site 7
Human Chromosome 7q32.2
