# Supplementary material for: Glycogen synthase kinase 3 beta inhibits microRNA-183-96-182 cluster via the β-Catenin/TCF/LEF-1 pathway in gastric cancer cells
Source: Nucleic Acids Res. 2013 Dec 12;42(5):2988–98. doi: 10.1093/nar/gkt1275 (PMC3950676; doi:10.1093/nar/gkt1275)
Supplement: Supplementary Data [file supp_gkt1275_nar-02232-y-2013-File010.docx]

Supplementary Table S1 Primers used in the experiments

| Primer name | Sequence |
| --- | --- |
| hmir183F | CCG CAG AGT GTG ACT CCT GTT CT |
| hmir183R | TCG TGG ATC TGT CTC TGC TCT GTT TAT G |
| hmir182F | GAG CTG CTT GCC TCC CCC C |
| hmir182R | GTG CCG GCT GAG TCC TCG C |
| hmir96F | TGG CCG ATT TTG GCA CTA GCA CA |
| hmir96R | TTT CCC ATA TTG GCA CTG CAC ATG ATT G |
| hm48129F | GAC AGC GGG TCT TTC CTA AAC CT |
| hm48154F | CCA AGC GTC GAG GAA CCC AA |
| hm50944R | CCG GCC CCA GAC ACT CAC C |
| hm51079R | CGC GTC ATC CCC GAA AGC C |
| hm51811R | CCT CGG CCC TAA TCG CTC AGA |
| hm52253R | CAG AGG TCC CGC TAA CTC CC |
| 18S-H-F | GTA ACC CGT TGA ACC CCA TT |
| 18S-H-R | CCA TCC AAT CGG TAG TAG CG |
| ACTB-H-F | CAC TCT TCC AGC CTT CCT TC |
| ACTB-H-R | GGA TGT CCA CGT CAC ACT TC |
| GAPDH-H-F | ATC ATC CCT GCC TCT ACT GG |
| GAPDH-H-R | GTC AGG TCC ACC ACT GAC AC |
| hmir193bF | GTG GTC TCA GAA TCG GGG TTT TGA GG |
| hmir193bR | ATG ACC CCA AAA GCG GGA CTT TGA |
| hm48154FSAC | CCC CCC CCG AGC TCC AAG CGT CGA GGA ACC CAA |
| hm48605F | TTC TCG ACT TTC AGC AGC GCA TT |
| hm48605FSAC | CCC CCC CCG AGC TCT TCT CGA CTT TCA GCA GCG |
| hm50944RHND | CCC CCC CCA AGC TTC CGG CCC CAG ACA CTC ACC |
| 17634f | GAT GAG AAG AAA GGA TCC AG |
| 17790r | TGG TGC CGT AGA TAC TAA CAT |
| 17790f | ATG TTA GTA TCT ACG GCA CCA |
| 17960r | CGC CAG TCT TGA GCA C |
| 17960f | GTG CTC AAG ACT GGC G |
| 18038r | CGA ACG CAA AGT GTC C |
| 17990F | CTT CTC AAA AGT CTA GAG CCA |
| 18105R | TTC AGT CAG GAG CTT ACC C |
| 52274F17 | AGT CGG CCT TAC GCA AA |
| 52433R17 | AGC TCC TTT CCC CGA AG |
| 52072F21 | TCT GTC CTA GTG GCC TCA TCG |
| 52168R16 | CCC AGG TCC GAA CCG A |
| 51838F17 | GTG CCG TAA TCG ACC TC |
| 51941R16 | CGA GAA TCC GCC ACC T |
| 51702F17 | CGA TTC CAC GGT GTC CT |
| 51812R18 | CTC GGC CCT AAT CGC TCA |
| 51463F17 | TCT CAG ACC TCC CGT GT |
| 51522R17 | AAC GGT GTC TCA TTC GC |
| 51033F16 | GGC CGC ACT AGA ACC C |
| 51079R16 | CGT CAT CCC CGA AAG C |
| DC2057F | CAC ATG CCG CAT CCA CAT |
| DC2057R | TAG GTA TGG TGT GGT GCT TG |
| DC2172F | CAC ACA CAC ACA CAC ACA CG |
| DC2172R | GGG GAG AGA ATG GGA GGA G |
| DC801F | CAC ATG ACT CCT CCC CCT TA |
| DC801R | TCC TGC ATT GAG TCG TTC TG |
| HM96F | GCC ATC TGC TTG GCC GAT TTT G |
| HM96R | GCT GCG GGT CCT GCT TTT C |
| HM96FERI | CCG GAA TTC GCC ATC TGC TTG GCC GAT TTT G |
| HM96RKPN | CGG GGT ACC GCT GCG GGT CCT GCT TTT C |
| HM182F | GCT CCT GGG GGG AGC TGC |
| HM182R | CTT CCC TCG CTG GCT GTG C |
| HM182FERI | CCG GAA TTC GCT CCT GGG GGG AGC TGC |
| HM182RKPN | CGG GGT ACC TGC ACA GGG TGC CGG CT |
| HM183F | GGC AGG CCG CAG AGT GTG AC |
| HM183R | GGT AAG GTG CTC CGG AGG CCC |
| HM183FXBA | TGC TCT AGA GGC AGG CCG CAG AGT GTG AC |
| HM183RKPN | CGG GGT ACC GGT AAG GTG CTC CGG AGG CCC |
| 49647F | AGG CCA CCC CAA CGC TGA CA |
| 49763R | CAG CCG GGG CTG GGG C |
| 49842F | AGG GGG AGA TGG GAA CGG GTC TG |
| 49969R | TGG AAT GGG GAC AAG GGG CCT |
| 50025F | GAG ACA GGT CAG CCT TGA GCA CCT T |
| 50121R | GTG CTT GGA AAT CCG ACC CTG GGA G |
| 50213F | CCT CGG CTA GCG GCA CCC T |
| 50300R | CCC CGC CTG CTT GCC CAA C |
| 50431F | CAC CTG AGC CAA TGG CGC TGC |
| 50537R | CTG CGG CTC CGG CAC GG |
| 50594F | CTG TTC TTC CGG TCC TCG GGC |
| 50695R | CTG GGT GTC GGG TGA CAG GTC TG |
| 50828F | TCA AGC TCC GTC CAT GCG GCT C |
| 50935R | CAG ACA CTC ACC TGC GCC GG |

Supplementary Method

**In silico screening of TCF/LEF-1 binding site (TBE) in the promoter of miR-183-96-182 cluster gene.** We first downloaded the gene sequence from GenBank, then identified the locations of mature miR-183, miR-96 and miR-183. We screened -10k bp upstream of the first nucleotide of miR-96 using TBE binding motifs AACAG, CAAAG, AAAAG or CACAG. We found the seven binding sites as shown in Figure 5A. We further confirmed the binding of TBE to these sites by ChIP assay. We also confirmed that the binding was functional by luciferase assay.

Supplementary Figure S1. Transcription factors that potentially regulate miR-183-96-182 cluster gene.
